# A Novel Technique of Impression Procedure in a Hemimaxillectomy Patient with Microstomia

**DOI:** 10.1155/2012/272161

**Published:** 2012-12-18

**Authors:** Suryakant C. Deogade

**Affiliations:** Department of Prosthodontics, Hitkarini Dental College & Hospital, Jabalpur, Madhya Pradesh 482005, India

## Abstract

A restricted mouth opening in hemimaxillectomy patient can create a significant problem with the insertion and the removal of the obturator prosthesis. Even it poses a problem in impression making due to small oral opening. A modification of the standard impression procedure is often necessary to accomplish an acceptable impression in the fabrication of a successful prosthesis. Sectional trays are a good option for such patients. This paper describes a novel technique of impression procedure and a method of fabricating a sectional tray with the anterior and the posterior locking mechanism for a hemimaxillectomy patient with limited oral opening.

## 1. Introduction

Patients with extensive head and neck injuries due to trauma and/or extensive surgical procedures often exhibit a severely limited ability to open the mouth. For the dentist involved in prosthodontic treatment of such patients, restricted maximal opening commonly leads to compromised impressions and prostheses. In prosthodontic treatment, the loaded impression tray is the largest item requiring intraoral placement. During impression procedures, wide mouth opening is required for proper tray insertion and alignment. Because this is not possible in patients with restricted opening ability, a modification of the standard impression procedure is often necessary to accomplish this fundamental step in the fabrication of a successful prosthesis [1].

Microstomia is defined as an abnormally small oral orifice [2]. Other causes of microstomia are scleroderma, oral submucous fibrosis, sequelae of burns, genetic disorders, Plummer Vinson's syndrome, surgical resection of facial and oral neoplasms, and temporomandibular joint disorders [3–6]. Patients with microstomia due to pathology or extensive surgical procedures often exhibit severely limited ability to open the mouth. As the size of the oral opening decreases, the difficulty in the planned treatment procedures also increases. 

The reduced mouth opening hinders conventional dental treatment; hence, alternative treatment procedures have to be chosen to overcome the clinical difficulties while managing such a patient. Several stock and custom tray designs have been described in the literature. Sectional impression trays have been fabricated using, orthodontic expansion screws [7], metal pins and acrylic resin block [8], lego blocks [9], dowel plug holes and a screw joint for rigid connection [10], locking levers [11], and interlocking tray segments [12]. Flexible impression tray with silicone putty has been also used in microstomia patient [13].

This paper presents a novel modification of previously described methods used in the fabrication of custom sectional tray with additional anterior and posterior locking mechanism by using press buttons and acrylic resin blocks. It also describes an alternative impression procedure for the prosthetic rehabilitation of a hemimaxillectomy patient with limited mouth opening.

## 2. Clinical Report

A 70-year-old man visited the Department of Prosthodontics, Hitkarini dental college and hospital, Jabalpur (India), for the fabrication of definitive obturator prosthesis. He underwent right hemimaxillectomy procedure to treat squamous cell carcinoma. The patient presented with an obvious and typical nasal twang, and he was experiencing difficulty in speech and deglutition. 

**Figure 1:**
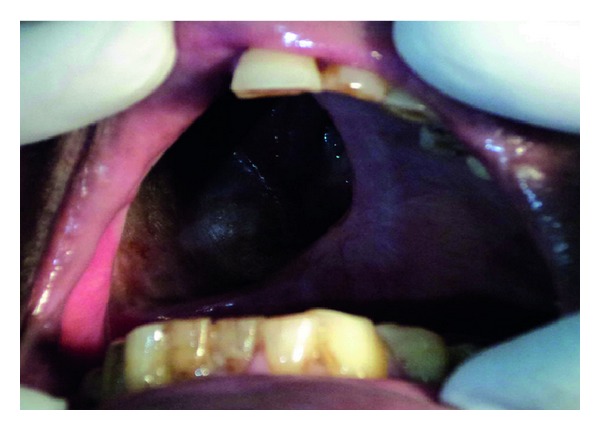
Intraoral view of defect.

**Figure 2:**
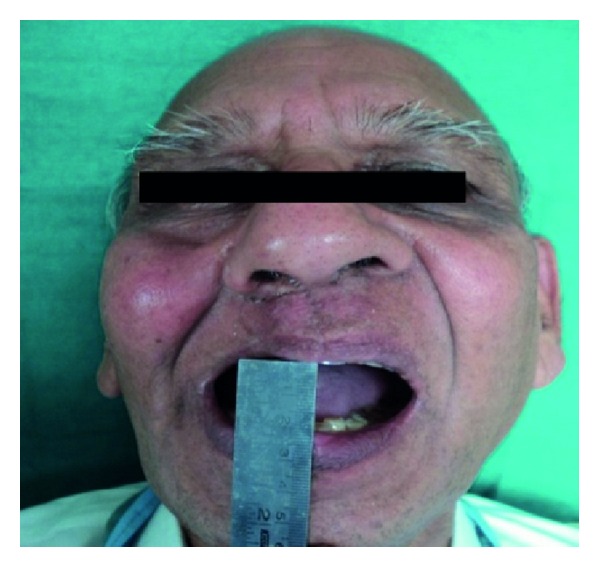
Restricted mouth opening.

Intraoral examination revealed a large but well-healed Armany's class I defect [14] on the right side of the maxilla along with loss of dentition on the same side (Figure 1). Patient had a severe restricted mouth opening of 18–20 mm due to postsurgical scar formation and radiation therapy (Figure 2). Hence, the decision was made to fabricate a custom sectional tray for definitive impression procedures.

## 3. Preliminary Impression

**Figure 3:**
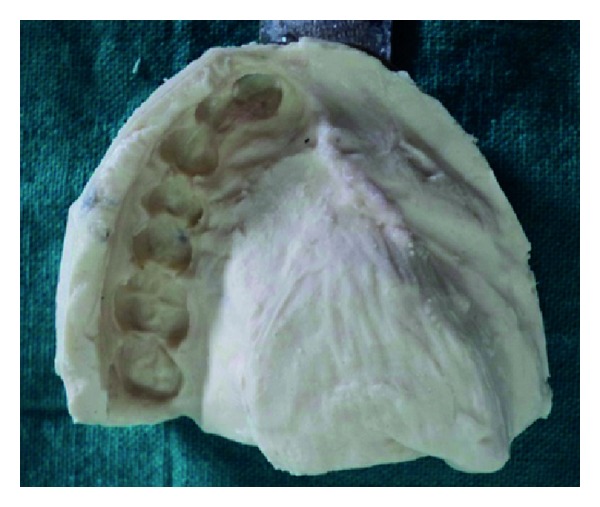
Primary impression.

Because of limited mouth opening, the suitable perforated stock tray was selected whose flange on the defect side was shortened until it could be inserted in patient's mouth, and the impression was made with irreversible hydrocolloid (Dentalgin; Prime Dental Products, Mumbai, India) (Figure 3). 

**Figure 4:**
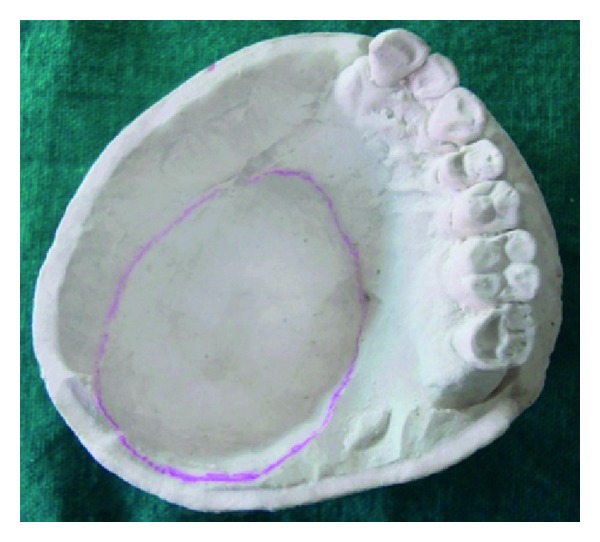
Primary cast.

 After retrieval of the tray, the cast was poured with type II gypsum material (Figure 4). After that, wax spacers (modeling wax; Deepti Dental Products, Ratnagiri, India) were adapted such that there were four tissue stops in each section to stabilize the tray when used in sections in maxillary arch.

## 4. Fabrication of Custom Sectional Tray

**Figure 5:**
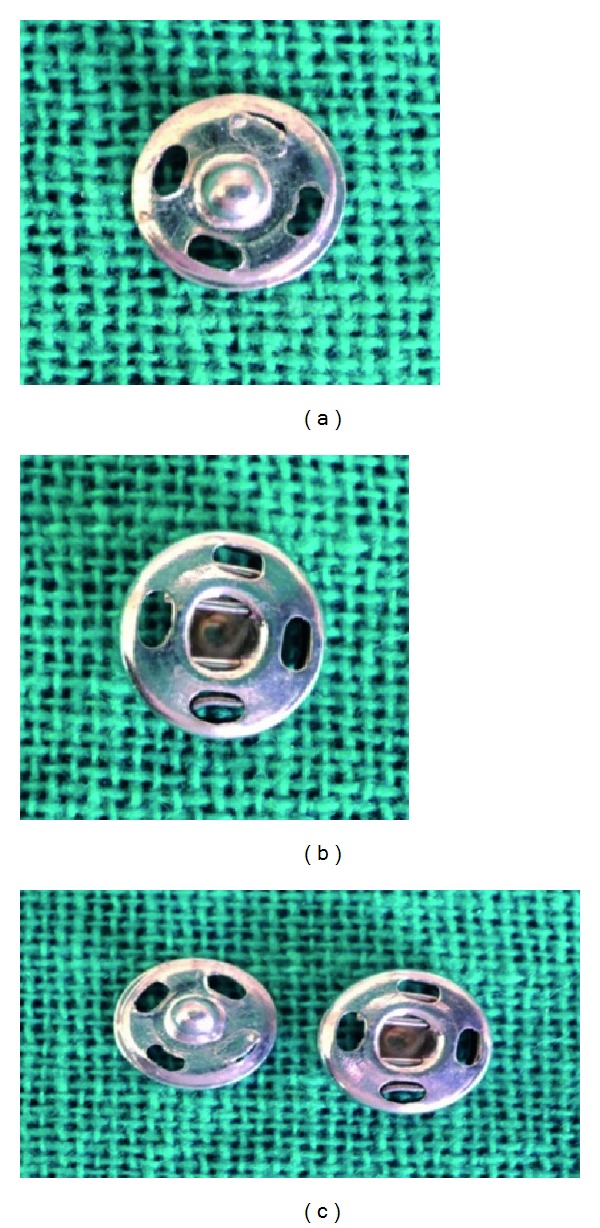
(a) Male button, (b) Female button, (c) Press button.

Acrylic resin blocks are used for the fabrication of the handle of the tray, whereas the press buttons (press button; Needle industries India Pvt. Ltd., Nilgiris, Tamilnadu) are used as the locks. Press buttons have a male and female parts (Figures 5(a), 5(b), and 5(c)). These buttons are commercially available. The handle and the press button functions as an anterior and posterior locks. 

**Figure 6:**
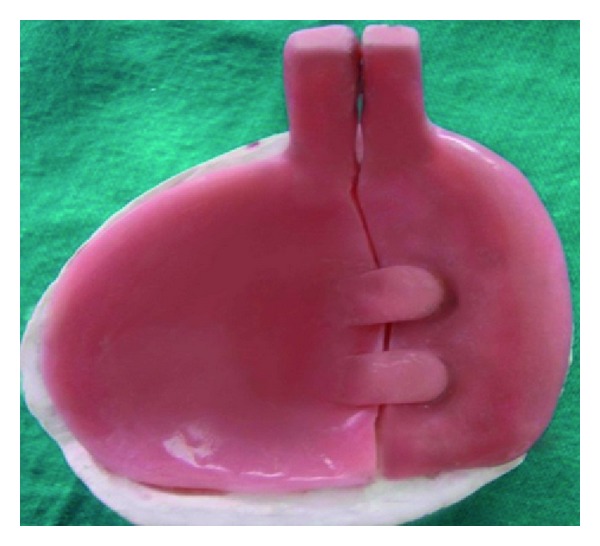
Custom sectional tray.

**Figure 7:**
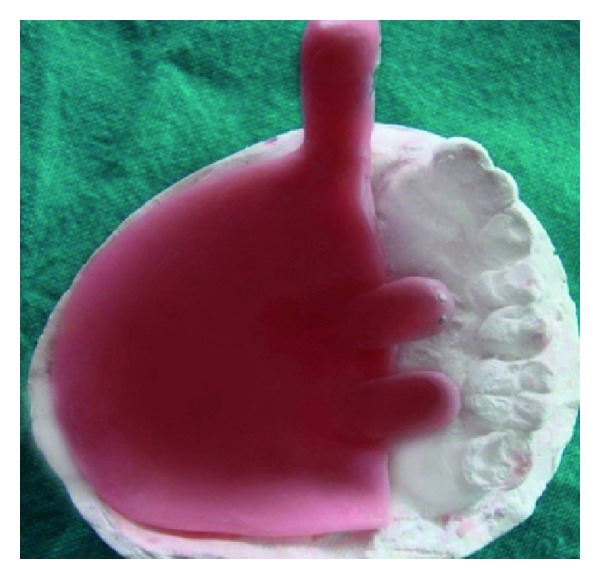
Male part.

**Figure 8:**
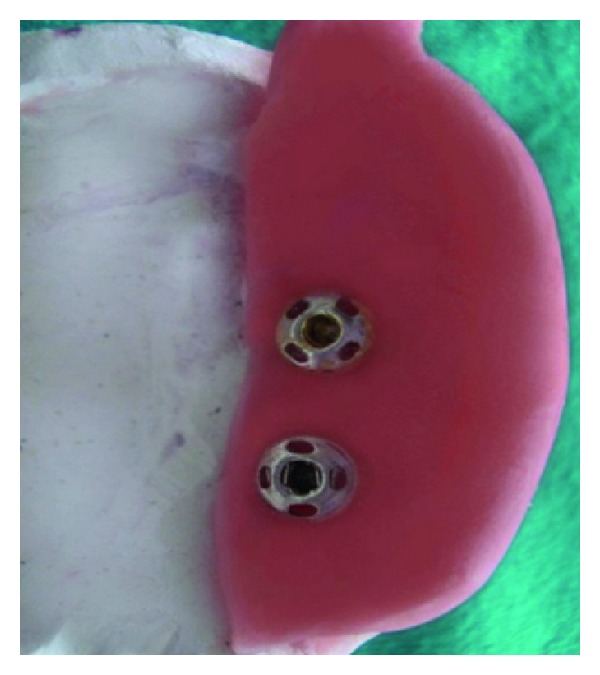
Female part.

**Figure 9:**
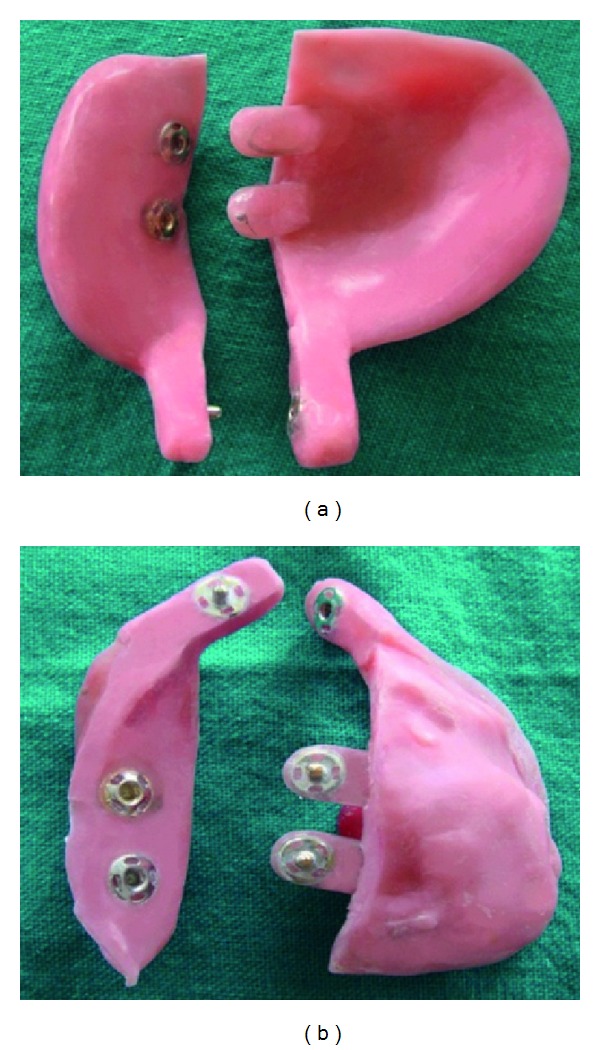
(a) and (b) Showing anterior and posterior locking mechanism.

**Figure 10:**
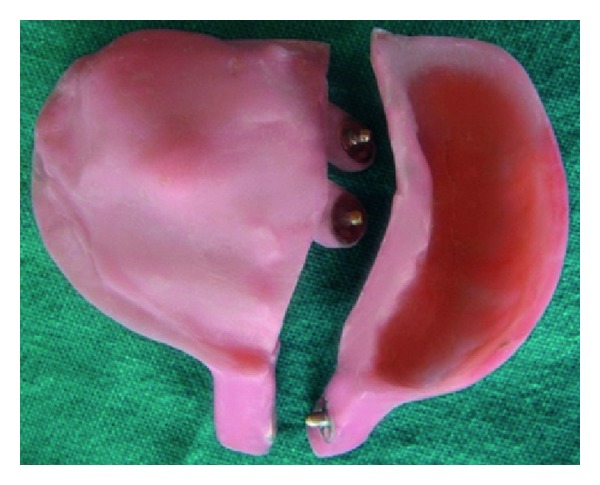
Dissembled sectional tray.

**Figure 11:**
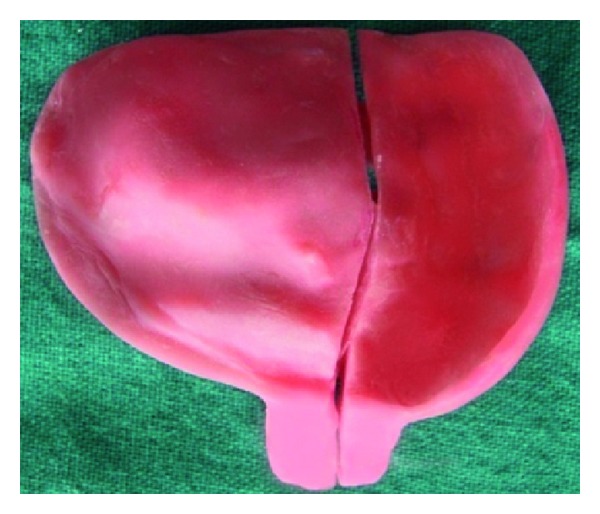
Assembled sectional tray.

The sectional tray was designed into right and left section (Figures 6, 7, 8 and 9). These sections could be detached and then joined together in the correct original position with the help of snap fit buttons (Figures 10 and 11).

Autopolymerizing acrylic resin (DPI cold cure; Dental Products of India, Mumbai, India) was used for the fabrication of the tray. After removing the wax spacers, impressions of each half of the arches were carried out separately. 

## 5. Definitive Impression

A medium and light viscosity poly (vinyl siloxane) elastomeric impression material (Reprosil; Dentsply DeTrey GmbH, Konstanz, Germany) is used to minimize errors due to manipulation distortion after setting. In order to make a definitive impression, and following step-by-step procedure is performed.

**Figure 12:**
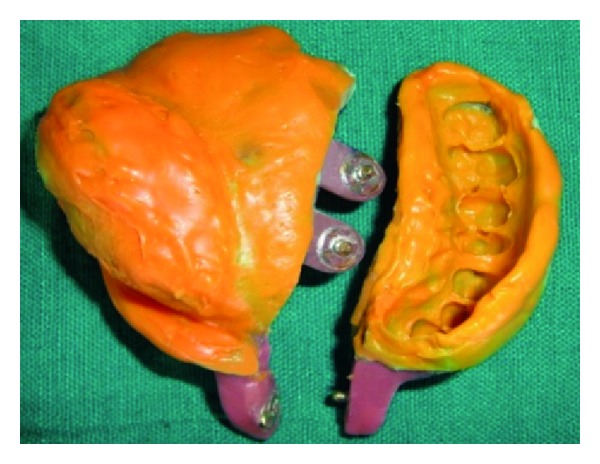
Dissembled impression.

**Figure 13:**
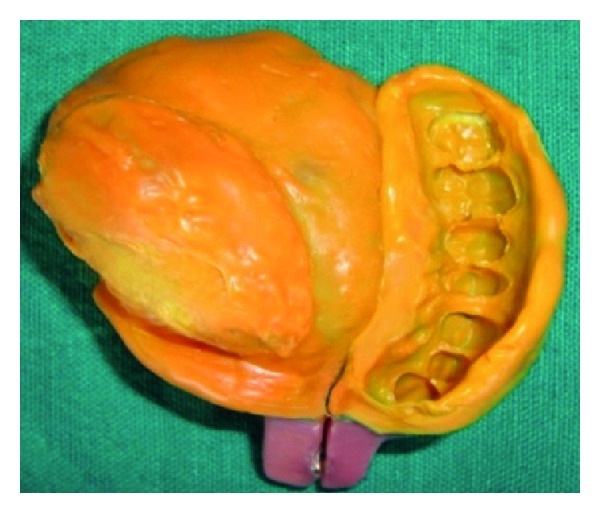
Assembled impression.

**Figure 14:**
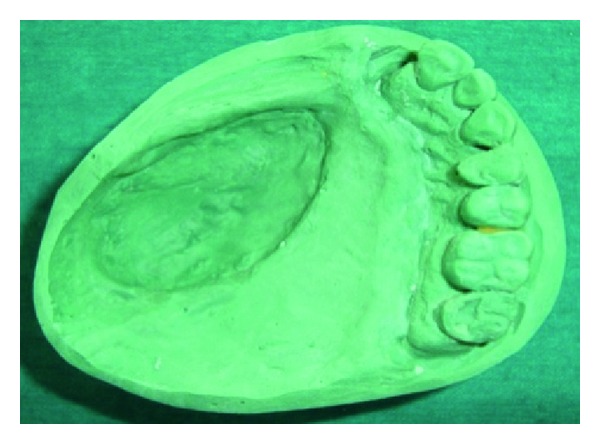
Master cast.

1. Make an impression with the first half of the tray. After removing it from the patient's mouth, trim the impression material so that it flush with the medial edge of the tray.
2. For the parts of the impression tray or material that will contact the second half tray, lubricate them and reinsert the tray in the mouth.
3. Load the second tray half with impression material and insert it in the mouth. Squeeze together the two tray parts at the handle. After ensuring precise fit, snap fit buttons are pressed firmly and allow the material to set.
4. Unpress the snap fit buttons and remove the parts individually (Figure 12).
5. Reassemble the tray outside the mouth; sticky wax or modeling plastic may be placed across the external tray component joints to stabilize the tray assembly (Figure 13).
6. Conventional prosthodontic protocols of boxing and pouring the impression were used with type III gypsum material (Kalstone; Kalabhai Karson, Mumbai, India) to create a definitive cast (Figure 14).

## 6. Discussion 

 The prosthodontist plays an important role in the rehabilitation of maxillofacial defects with limited mouth opening. A method of overcoming impression difficulties that uses a sectional custom impression tray that results in an accurate impression for such patients is described.

In the present case, mouth opening was about 18 mm which posed a significant problem while making impressions. The patient was treated for squamous cell carcinoma and was having the classical Armany's class I defect. As the resection bed was treated postoperatively with radiation therapy, it resulted in limited oral opening.

Limited mouth opening often complicates and compromises the treatment of patients. Sectional trays are an alternative treatments in such conditions [11]. One of the requirements of the sectional trays are the ease of reassembling and disassembling the tray in the mouth. This feature necessitated to incorporate an easy locking mechanism [10]. It is observed that in the absence of a posterior lock, the tray would separate in the posterior palatal seal region making the impression difficult. This realized the need of using posterior lock in the maxillary sectional tray, as in the current design. 

Hence, the decision was taken to fabricate the sectional tray with the help of readily available options like acrylic blocks and snap fit buttons. The trays could be detached and reattached again which made a very good option for the patient. Advantages of the technique include very economical simplified tray manipulation and decreased patient trauma, the ability to use a custom fabricated tray for optimal impression material thickness, and precise intraoral positioning and stability. Disadvantages are the additional time, materials, and labor required for precise fabrication of the sectional tray and secondary impression and the requirement for correct fitting of the components to produce an accurate cast.

## 7. Conclusion

Restricted mouth opening in hemimaxillectomy patients is a common scenario due to postsurgical scar formation and radiation therapy. Making good impressions is an important step in prosthodontic management of such patients. Keeping this in mind, we have to modify the routinely used trays and provide newly designed trays for ease and betterment. This paper describes very simple, quick, economical, and readily available methods of dealing with patients in whom placement of full size impression tray is hindered by microstomia.

Alignment and stability of a reassembled sectional tray is necessary, and it can be achieved by using an anterior and posterior lock. These features are present in the current design that helps to overcome difficulties while making impressions in such patients.
